# Prevalence of Subclinical Hypothyroidism in Pregnant Women in Tehran-Iran

**Published:** 2014-07-08

**Authors:** Fakhrolmolouk Yassaee, Masoumeh Farahani, Ali Reza Abadi

**Affiliations:** 1 Department of Obstetrics and Gynecology, Taleghani University Hospital, Shahid Beheshti University of Medical Sciences, Tehran, Iran; 2 Department of Obstetrics and Gynecology, Shahid Beheshti University of Medical Sciences, Genomic Research Center, Infertility and Health Reseach Center, Tehran, Iran; 3 Department of Community Medicine, Faculty of Medicine, Shahid Beheshti University of Medical Sciences, Tehran, Iran

**Keywords:** Hypothyroidism, Pregnancy, Prevalence, Thyrotropin

## Abstract

**Background:**

Maternal subclinical hypothyroidism during pregnancy is associated with various adverse outcomes. Recent consensus guidelines advocate universal thyroid function screening during pregnancy. There are no data from Iran about the prevalence of thyroid hypofunction in pregnancy. This study aims to find the prevalence of thyroid dysfunction.

**Materials and Methods:**

In this descriptive cross sectional study, thyrotropin (TSH) was measured in 3158 pregnant women irrespective of gestational age from October 2008-March 2012. If TSH was more than 2.5 mIU/L in the first trimester or more than 3 mIU/L in the second or third trimester, free T4 was measured to diagnose subclinical/ overt hypothyroidism. If serum free T4 was in the normal range (0.7-1.8 ng/dl) the diagnosis was subclinical hypothyroidism and if below the normal range, overt hypothyroidism was diagnosed.

**Results:**

A total of 3158 pregnant women were evaluated. One hundred forty seven of them were diagnosed as hypothyroidism. Subclinical hypothyroidism and overt hypothyroidism were present in 131 (89.1%) and 16 (10.9%) women respectively. Prevalence of subclinical hypothyroidism was 4.15%. Most of the subclinical and overt hypothyroidism cases were diagnosed in the first trimester.

**Conclusion:**

It appears logical to check TSH during pregnancy due to the observed prevalence of subclinical hypothyroidism.

## Introduction

Thyroid disorders are among the common endocrine problems in pregnant women. It is now well known that not only overt, but also subclinical hypothyroidism (SCH) has adverse effects on maternal and fetal outcome (1). There are studies which show low intellectual and motor development of children is associated with abnormalities of maternal thyroid function (1, 2). According to the study by Sahu et al. in India on 633 pregnant women, the prevalence of subclinical and overt hypothyroidism was 6.5% and 4.6% respectively (3). Fetal thyroid gland is not functional up to 12 weeks of gestation. Thyroid releasing hormone crosses the placenta to stimulate fetal thyroid. So maternal thyroid function is very important during the first trimester (4). During the first trimester, human chorionic gonadotropin (hCG) level is high that act like thyrotropin (TSH) (α subunit of hCG and TSH is similar). So the range of TSH, under the influence of placental hCG, is decreased during pregnancy with the lower normal TSH level in the first trimester being poorly defined and an upper limit of 2.5 mIU/L. At 10-12 weeks of gestation, plasma level of hCG begin to decline to act like TSH, so TSH is increased a little to an upper normal limit of 3mIU/L in the second and third trimester (4). According to the study by Soldin et al. trimester-specific measurements of T3, FT4 and TSH is warranted (5). However, a study in Iran by Zarghami et al. stated that TSH level did not show significant differences in different trimesters of pregnancy(6). There is no data from Iran about the prevalence of SCH in pregnancy and there is debate about universal screening of thyroid function in pregnancy.

We therefore studied the thyroid function of pregnant women to know the prevalence of subclinical hypothyroidism.

## Materials and Methods

This descriptive cross-sectional study was done on 3158 pregnant women in Taleghani Hospital in Tehran, Shahid Beheshti University of Medical Sciences from October 2008-March 2012. For all pregnant women in the first prenatal care and during routine laboratory workup, screening of thyroid function was done by TSH level in endocrine laboratory in Taleghani Hospital by the chemiluminescent immunoassay (Elecsys 2010, Hitachi, Diamond, Japan). If TSH level was >2.5 mIU/L in the first trimester or TSH >3 mIU/L in the second or third trimester, free T4 measurement was done by chemiluminescent immunoassay to know whether it is subclinical or overt hypothyroidism. If serum FT4 was in the normal range (0.8-1.7 ng/dl) SCH was diagnosed and if below the normal range, OH was the diagnosis.

Their demographic (maternal age, gestational age, parity) and clinical details were collected as part of routine antenatal care and were recorded. We asked the women about personal and family history of thyroid disease. Duration of gestation was calculated from last menstrual period and verified by ultrasonography. The Ethical Committee of Shahid Beheshti University of Medical Sciences approved this study and informed consent was taken from all participants. SPSS software version 20 were used for data analysis including t test.

## Results

The age of patients ranged from 17-38 years old with mean ± SD (27 ± 5) (Table 1). Among 3158 women, 147 were diagnosed as hypothyroidism (Table 2). Eighty-four (57.1%) were nulliparous and 63 (42.9%) were multiparous.

**Table 1:** Distribution of patient age of overt and subclinical hypothyroidism in pregnant women in Tehran between 2008 and 2012


Age(Y)	Frequency	Percent	Cumulative frequency

**<20**	18	12.2	12.2
**20-25**	36	24.5	36.7
**25-30**	61	41.5	78.2
**>30**	32	21.8	100
**Total**	147		


**Table 2:** Prevalence of overt and subclicinal hypothyroidism in pregnant women in Tehran (2008-2012)


Type of hypothyroidism	No.of patients	Prevalence

**Subclinical**	131(89.1%)	4.15%
**Overt**	16(10.9%)	0.5%
**Total**	147(100%)	4.65%


Most of the women with subclinical hypothyroidism were diagnosed in the first trimester and many women were undetected till the second and third trimesters (Table 3).

According to table 3 there was a significant difference in mean age (about 3.5 years) of patients in overt and subclinical groups (p=0.02) .This means that universal screening is necessary because most of the pregnancies occur in young women.

**Table 3:** Relationship between mean age and gestational age and type of hypothyroidism in pregnant women in Tehran (2008-2012)


Diagnosis	Age(Y)		Number of patients in each trimester			
	Mean	SD	First	Second	Third	Total

**Subclinical**	26.85	4.989	76	31	24	131
**Overt**	30.38	4.856	9	4	3	16
**Total**	27.24	5.079	85	35	27	147


## Discussion

We found the prevalence of subclinical hypothyroidism to be 4.15%. According to the study by Casey et al. the prevalence of subclinical hypothyroidism during early pregnancy is common, affecting about 2.5% pregnant women (7, 8). A similar result was reported by Allan et al. (9), Vaidya et al. (10) and Mannisto et al. (11). These studies are in contrast with the report by Gillett who stated that routine screening of pregnant women is not necessary for thyroid function, unless they were at increased risk of thyroid disease (12).

This suggests that subclinical hypothyroidism is more common in Iranian pregnant women. Subclinical hypothyroidism during early pregnancy has been shown to be associated with impaired neuropsychological development of children and several other adverse outcomes, including preterm delivery, preeclampsia and increased fetal mortality (1-4, 8, 10, 11, 13-16). But the study by Cleary Goldman et al. showed that subclinical hypothyroidism is detectable in 2.2% in the first and second trimesters with no adverse outcome in pregnant women with thyroid hypofunction (15). Pregnancy has much influence on the thyroid gland and thyroid function. Physiological changes of pregnancy cause the thyroid gland to increase production of thyroid hormones to meet maternal and fetal needs. TSH and human chorionic gonadotropin (hCG) have identical α subunits whereas the β subunits differ in their amino acid sequence (4). There is also an uncertainty regarding the most appropriate initial screening test for thyroid dysfunction in pregnancy. The consensus guidelines recommend using TSH level as the initial test (10, 14-16). The American College of Obstetricians and Gynecologists (2007) concluded that although observational data were consistent with the possibility that subclinical hypothyroidism was associated with adverse neuropsychological development, there have been no interventional trials to demonstrate improvement in decision to do routine thyroid screening of pregnant women.There are reports that testing the high risk group only for thyroid function would miss about one third of pregnant women with overt/subclinical hypothyroidism (7, 9, 17-22). Most of our patients with overt hypothyroidism were diagnosed in the first trimester. This is in agreement with a previous study by Sahu et al. in India in which the rate of overt hypothyroidism was reported as 4.6% (3). We know that patients with overt hypothyrodism usually are infertile and if they become pregnant, complications of pregnancy such as abortion may occur. So universal screening for thyroid function appears logical. Also, diagnosis of subclinical hypothyroidism during the third trimester is necessary to treat them and prevent postpartum depression.

## Conclusion

There is a high percentage of pregnant women that reach second and third trimester of pregnancy with undiagnosed thyroid disease. It is therefore necessary to screen women with a serum TSH, if they are pregnant or deciding to become pregnant.

## References

[1] Haddow JE, Palomaki GE, Allan WC, Williams JR, Knight GJ, Gagnon J (1999). Maternal thyroid deficiency during pregnancy and subsequent neuropsychological development of the child. N Engl J Med.

[2] Li Y, Shan Z, Teng W, Y u X, Li Y, Fan C (2010). Abnormalities of maternal thyroid function during pregnancy affect neuropsychological development of their children at 25-30 months. Clin Endocrinol (Oxf).

[3] Sahu MT, Das V, Mittal S, Agrawal A, Sahu M (2010). Overt and subclinical thyroid dysfunction among Indian pregnant women and its effect on maternal and fetal outcome. Arch Gynecol Obstet.

[4] Stagnaro-Green A, Abalovich M, Alexander E, Azizi F, Mestman J, Negro R (2011). Guidelines of the American Thyroid Association for the diagnosis and management of thyroid disease during pregnancy and postpartum. Thyroid.

[5] Soldin OP, Tractenberg RE, Hollowell JG, Jonklaas J, Janicic N, Soldin SJ (2004). Trimester-specific changes in maternal thyroid hormone,thyrotropin, and thyroglobulin concentrations during gestation: trends and associations across trimesters in iodine sufficiency. Thyroid.

[6] Zarghami N, Rohbani-Noubar M, Khosrowbeygi A (2005). Thyroid hormones status during pregnancy in normal Iranian women. Indian J Clin Biochem.

[7] Casey BM, Dashe JS, Sponge CY, McIntire DD, Leveno KJ, Cunningham GF (2007). Perinatal significance of isolated maternal hypothyroxinemia identified in the first half of pregnancy. Obstet Gynecol.

[8] Casey BM, Dashe JS, Wells CE, McIntire DD, Byrd W, Leveno KJ (2005). Subclinical hypothyroidism and pregnancy outcomes. Obstet Gynecol.

[9] Allan WC, Haddow JE, Palomaki GE, Williams JR, Mitchell ML, Hermos RJ (2000). Maternal thyroid deficiency and pregnancy complications: implications for population screening. J Med Screen.

[10] Vaidya B, Anthony S, Bilous M, Shields B, Drury J, Hutchison S (2007). Detection of thyroid dysfunction in early pregnancy: Universal screening or targeted high risk case finding?. J Clin Endocrinol Metab.

[11] Männistö T, Vääräsmäki M, Pouta A, Hartikainen AL, Ruokonen A, Surcel HM (2009). Perinatal outcome of children born to mothers with thyroid dysfunction or a antibodies: a prospective population-based cohort study. J Clin Endocrinol Metab.

[12] Gillett M (2004). Subclinical hypothyroidism: Subclinical thyroid disease: Scientific review and guidelines for diagnosis and management. Clin Biochem Rev.

[13] Abalovich M, Gutierrez S, Alcaraz G, Maccallini G, Garcia A, Levalle O (2002). Overt and subclinical hypothyroidism complicating pregnancy. Thyroid.

[14] Kourtis A, Makedou K, Giomisi A, Mouzaki M, Slavakis A, Kalogiannidis L (2010). Prevalence of undiagnosed thyroid disease in pregnancy. Endocrine Abstracts.

[15] Cleary-Goldman J, Malone FD, Lambert-Messerlian G, Sullivan L, Canick J, Porter TF (2008). Maternal thyroid hypo function and pregnancy outcome. Obstet Gynecol.

[16] Pop VJ, Brouwers EP, Vader HL, Vulsma T, Van Baar AL, de Vijlder JJ (2003). Maternal hypothyroxinemia during early pregnancy and subsequent child development: a 3-year follow-up study. Clin Endocrinol(Oxf).

[17] Negro R, Schwartz A, Gismondi R, Tinelli A, Mangieri T, Stagnaro-Green A (2010). Universal screening versus case finding for detection and treatment of thyroid hormonal dysfunction during pregnancy. J Clin Endocrinol Metab.

[18] McLeod DSA, McIntyre HD (2010). Subclinical hypothyroidism and related biochemical entities in pregnancy :implications and management. Obstet Med.

[19] Davis LE, Leveno KJ, Cunningham FG (1988). Hypothyroidism complicating pregnancy. Obstet Gynecol.

[20] Benhadi N, Wiersing WM, Reitsma JB, Vrijkotte TG, Bonsel GJ (2009). Higher maternal TSH levels in pregnancy are associated with increased risk for miscarriage, fetal or neonatal death. Eur J Endocrinol.

[21] Wang W, Teng W, Shan Z, Wang S, Li J, Zhu L (2011). The prevalence of thyroid disorders during early pregnancy in China: the benefits of universal screening in the first trimester of pregnancy. Eur J Endocrinol.

[22] Ashoor G, Maiz N, Rotas M, Jawdat F, Nicolaides KH (2010). Maternal thyroid function at 11 to 13 weeks of gestation and subsequent fetal death. Thyroid.

